# Vital Signs: Obesity Among Low-Income, Preschool-Aged Children — United States, 2008–2011

**Published:** 2013-08-09

**Authors:** Ashleigh L. May, Liping Pan, Bettylou Sherry, Heidi M. Blanck, Deborah Galuska, Karen Dalenius, Barbara Polhamus, Laura Kettel-Khan, Laurence M. Grummer-Strawn

**Affiliations:** Div of Nutrition, Physical Activity, and Obesity, National Center for Chronic Disease Prevention and Health Promotion, CDC

## Abstract

**Background:**

The prevalence of obesity among U.S. preschoolers has doubled in recent decades. Childhood obesity increases the risk for adult obesity and is associated with negative health consequences. Trends in the state-specific prevalence of obesity among low-income U.S. preschool children have not been examined since 2008. State-specific obesity prevalence surveillance helps determine the need for and impact of state and local obesity prevention strategies.

**Methods:**

Measured weight and height data from approximately 11.6 million low-income children aged 2–4 years from 40 states, the District of Columbia, and two U.S. territories who participated in the Pediatric Nutrition Surveillance System during 2008–2011 were used to estimate state obesity prevalence. Obesity was defined as having an age- and sex-specific body mass index ≥95th percentile, according to the 2000 CDC growth charts. Logistic regression models adjusted for age, sex, and race/ethnicity were used to examine trends in the state-specific obesity prevalence.

**Results:**

During 2008–2011, statistically significant downward trends in obesity prevalence were observed in 18 states and the U.S. Virgin Islands. Florida, Georgia, Missouri, New Jersey, South Dakota, and the U.S. Virgin Islands had the largest absolute decreases in obesity prevalence, each with a decrease of ≥1 percentage point. Twenty states and Puerto Rico experienced no significant change, and obesity prevalence increased significantly in three states.

**Conclusions and Implications for Public Health Practice:**

Small but significant declines in obesity among low-income preschoolers were observed in 19 of 43 states/territories examined. Continued prevention efforts are needed to sustain and expand the implementation and evaluation of population-level interventions to prevent childhood obesity.

## Introduction

Data from the National Health and Nutrition Examination Survey indicate that nationally the prevalence of obesity remains high among all youths, including preschool-aged children, however there was no statistically significant change in obesity prevalence between 2007–2008 and 2009–2010. During 2009–2010, the prevalence of obesity was 12.1% among U.S. children aged 2–5 years with higher rates among some subgroups including non-Hispanic black (18.9%) and Hispanic (16.2%) children (1). Childhood obesity is associated with negative physical and mental health consequences (2). Overweight or obese preschoolers are five times as likely as to become overweight or obese adults as their non-obese peers (3). Preventing obesity early in life is a public health priority to improve health across the lifespan.

National estimates of the prevalence of obesity describe the extent of the problem for the U.S. population. However, state-specific obesity prevalence estimates provide opportunities for state and local health departments to monitor progress in controlling and preventing obesity. In addition, state-specific estimates are important because there is variability among states in demographic and other factors that might influence obesity and appropriate prevention strategies and decision-making related to programmatic efforts, environmental supports, and policies might differ.

Limited studies are available on state-specific trends in obesity prevalence among young children. The few published reports focus on older school-aged children and adolescents (4–6) and use parent/child reports of height and weight to calculate body mass index (BMI) (4,5). Other reports on young children (7,8)* have used data from CDC’s Pediatric Nutrition Surveillance System (PedNSS); one report showed that among 44 states/territories during 2003–2008, nine showed a significant decline in obesity, 24 showed significant increases, and 11 showed no statistically significant change (7). The prevalence of obesity among low-income preschool children remains high. In 2011, the prevalence of obesity for all contributors to the PedNSS was 14.4%, was approximately 4% points higher than in the early 1990s† and varied considerably among states/territories. This report extends earlier PedNSS analyses (7,8) by examining the state-specific prevalence and trends of obesity during 2008–2011.

## Methods

PedNSS is a state-based public health surveillance system that monitors the nutritional status of low-income children from birth through age 4 years. Data are primarily collected from participants in the Special Supplemental Nutrition Program for Women, Infants, and Children (WIC). About 50% of low-income children eligible for WIC are enrolled (9), but data from other programs such as the Early and Periodic Screening, Diagnosis and Treatment program, and the Maternal and Child Health Bureau Title V program are also included. Not all WIC agencies submitted data to the PedNSS during 2008–2011 (9). Data were collected from children and their caregivers during routine visits to local public health clinics approximately twice per year. Children’s heights and weights were directly measured by trained clinical staff using standardized protocols. All data were submitted electronically to CDC. One randomly selected clinical record per child, per year, was used in this study. Obesity was defined as having an age- and sex-specific BMI ≥95th percentile according to the 2000 CDC growth charts.§

This study included approximately 12.1 million children aged 2–4 years from 43 PedNSS contributors, including 40 states, the District of Columbia, and two U.S. territories (U.S. Virgin Islands and Puerto Rico) that consistently reported data annually to PedNSS and used consistent methodology during 2008–2011. Height, weight, and BMI data that were missing (n = 258,310; 2.1%), miscoded (n = 8,124; 0.1%), or biologically implausible¶ (n = 285,572; 2.4%; e.g., height-for-age z-score <−5.0 or >3.0, weight-for-age z-score <−4.0 or >5.0, or BMI-for-age and sex z-score <−4.0 or >5.0) were excluded from all analyses, leaving an analytic sample of 11,590,087 low-income preschool children. The average sample size among states/territories across all years of study ranged from 2,516 to 294,209.

The state-specific prevalence of obesity among low-income preschoolers during 2008–2011 was calculated. To examine trends in the state-specific obesity prevalence during the study period, logistic regression models were used, adjusted for age, sex, and race/ethnicity. Adjusted odds ratios (AORs) were calculated to determine the estimated annual change in odds of obesity. To determine whether changes in obesity prevalence could be explained by socioeconomic status of participants, a secondary analysis was conducted that included household income as part of the adjusted model. AORs were considered statistically significant at p<0.05. Compared with the 2008 study population, the 2011 study population was older and included a slightly smaller proportion of non-Hispanic whites and a slightly larger proportion of non-Hispanic blacks (Table 1).

## Results

**FIGURE 1:**
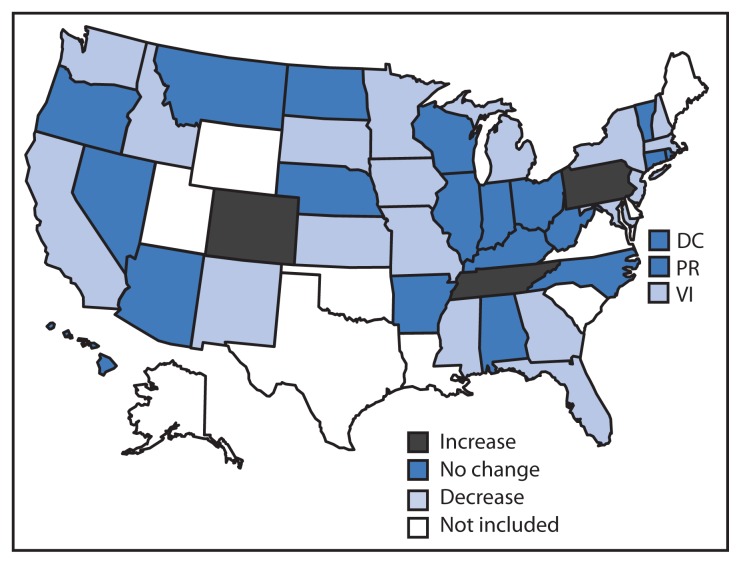
Decreases and increases^*†^ in obesity^§^ prevalence from 2008 to 2011 among low-income preschool-aged children — Pediatric Nutrition Surveillance System, United States ^*^ Trends assessed by logistic regression models adjusted for age, sex, and race/ethnicity. ^†^ Annual decreases and increases in obesity are statistically significant at p<0.05. ^§^ Obesity defined as having an age- and sex-specific body mass index ≥95th percentile, according to the 2000 CDC growth charts.

During 2008–2011, a total of 19 states/territories reported significant downward trends in obesity prevalence among low-income preschoolers (Figure 1). Among them, the largest decline in obesity prevalence was in the U.S. Virgin Islands (AOR = 0.92; CI = 0.87–0.97), where there was a decrease in the prevalence of obesity from 13.6% in 2008 to 11.0% in 2011, an absolute decrease of 2.6 percentage points (Table 2). In five additional states (Florida, Georgia, Missouri, New Jersey, and South Dakota) the absolute decrease in obesity prevalence from 2008 to 2011 was ≥1 percentage point. Across the 19 states/territories with significant downward trends, the absolute decrease in obesity prevalence from 2008 to 2011 ranged from 0.3 to 2.6 percentage points. The relative decreases in obesity prevalence among the 19 states/territories ranged from 1.8% to 19.1%.

An additional 21 states/territories experienced no significant trend in obesity prevalence. Three states experienced a significant upward trend in obesity prevalence. The absolute increase in obesity prevalence from 2008 to 2011 for the three states ranged from 0.6 to 0.7 percentage points. The relative increase in obesity prevalence among the three states ranged from 5.2% to 6.4%.

In a secondary analysis of the 34 states/territories that had complete data on household income, household income was added to the logistic regression model. Among these states/territories, significance changed for only one state. Montana went from no significant trend to a significant decrease in the prevalence of obesity (AOR = 0.97, CI = 0.94–1.00).

**FIGURE 2:**
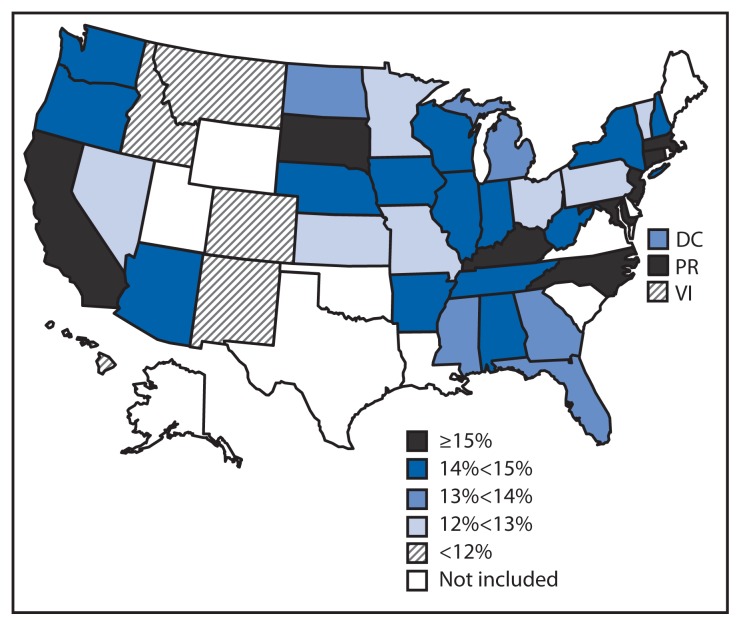
Prevalence of obesity among low-income, preschool-aged children — Pediatric Nutrition Surveillance System, United States, 2011 ^*^ Obesity defined as having an age- and sex-specific body mass index ≥95th percentile, according to the 2000 CDC growth charts.

In 2011, the prevalence of obesity among states/territories in the study ranged from 9.2% to 17.9% (Table 2). Ten states/territories had an obesity prevalence ≥15% (Figure 2), with the highest prevalence in Puerto Rico (17.9%). Six states/territories had an obesity prevalence <12%. The lowest obesity prevalence was in Hawaii (9.2%).

## Discussion

In recent years there have been small but significant decreases in childhood obesity. The finding that 19 states/territories experienced a decrease in obesity among low-income preschoolers, a vulnerable population, adds to recent findings from local data of low-income preschoolers (10,11) and studies observing decreases among children with higher socioeconomic status (SES) (12). The specific factors that might have contributed to the differential changes in obesity prevalence by state could not be readily identified. States likely differ by cultural and other factors that affect diet, activity, and weight as well as the implementation of policy and environmental interventions used to improve nutrition and physical activity. For example, reductions in obesity prevalence might reflect a combination of contributing factors, such as local and state initiatives that focus on the implementation of nutrition and physical activity standards for early care and education (ECE) programs (13) and efforts to improve healthier food options and physical activity offerings in communities (14). Federal policy changes such as the alignment of the WIC package of nutritious foods with the Dietary Guidelines for Americans (15) might have led to improved diets among low-income preschool children and their families. Population-level changes in behavior such as increases in breastfeeding (16) also might have contributed to declines in obesity.

During the same period, national initiatives such as Let’s Move,** reports such as the White House Childhood Obesity Task Force Report,†† and recommendations from groups including the National Resource Center for Health and Safety in Child Care and Early Education, American Public Health Association, American Academy of Pediatrics (13), and the Institute of Medicine (17) and media coverage have drawn attention to childhood obesity by building awareness and identifying stakeholders and potential actions to address the problem. Parents, schools, ECE providers, health-care providers, business leaders, the faith community, and state and local officials are among the groups identified in expert recommendations as stakeholders in the prevention of early childhood obesity. Each stakeholder makes decisions that influence the nutrition and physical activity environments where children live, learn, and play.

State and local officials have a unique opportunity to bring together various stakeholders concerned about children’s health. They can assist communities in conducting needs assessments, developing action plans, and launching initiatives aimed at increasing healthy eating and active living (18). State and local officials can support ECE providers by helping to identify and incorporate best practices for obesity prevention, including putting forth standards that promote healthy eating and physical activity (13), such as serving fruits and vegetables, limiting the regular consumption of sugary beverages (19), and providing more opportunities for physical activity, and reducing screen time. Officials also can help community groups and agencies identify or improve local play spaces to increase opportunities for physical activity (17,18) through efforts such as mapping, providing better access to recreational facilities, and promoting safety (19) in places where children can be physically active. Assisting local businesses and community groups in their support of families by educating them about healthful affordable food choices, providing point of decision prompts that promote healthy foods and beverages at the places they are purchased, providing opportunities for physical activity (17,18), increasing food store availability in underserved areas, and zoning for mixed land use (19) and improving access to safe, free drinking water in public places (18) are other ways that officials can help communities prevent childhood obesity. State and local officials also can engage communities in obesity prevention by leading or participating in coalitions (e.g., an obesity task force or food advisory council), and partnerships with groups in other sectors (18) like agriculture, retail, transportation, and economic development.

The findings in this report are subject to at least three limitations. First, PedNSS is limited to low-income children who participated in federal nutrition programs. These findings might not reflect the obesity prevalence and trends among all low-income U.S. preschool-aged children. In addition, the results might not be reflective of preschool-aged children of higher socioeconomic status who might have experienced more substantial declines in obesity prevalence (12). Second, this study included 43 states/territories that consistently collected PedNSS data during 2008–2011. Trends in other states/territories might differ. Finally, compared with 2008, the number of children in PedNSS was higher in subsequent years (approximately 2.7 million in 2008, 3.0 million in 2009, 3.0 million in 2010, and 2.9 million in 2011). This might have been caused, in part, by the economic downturn, which might have led to previously ineligible families becoming eligible for these nutrition programs. Whether or the degree to which these changes might have contributed to declines in obesity is unknown. As compared to the 2008 cohort, the 2011 cohort was slightly older and included a slightly smaller proportion of non-Hispanics whites and a slightly larger proportion of non-Hispanic blacks. Adjustments for these characteristics in logistical regression models might not have eliminated all confounding. Further adjustment for household income (for 34 states/territories with complete income data) however had little effect; one state went from no change to a significant decline.

Small decreases in the prevalence of obesity among low-income preschool children have been observed in certain states/territories. Continued prevention efforts remain necessary to ensure that this downward trend continues. State and local officials have an opportunity to lead these efforts through the continued development, implementation, and evaluation of obesity prevention initiatives and by leading partnerships with public and private sectors.

**Key Points**

- The prevalence of obesity declined slightly among low-income preschoolers in 19 of 43 states and territories during 2008–2011.
- Obesity rates are high among preschool children in the United States. Approximately one child in eight aged 2–5 years is obese.
- Overweight or obese preschoolers are five times as likely to become overweight or obese adults, compared with their normal weight peers. In older children and adolescents, obesity is associated with high cholesterol, high blood sugar, asthma, and mental health problems.
- To continue the downward trend in obesity, continued communitywide action is needed. State and local officials can help prevent obesity in young children by 1) creating partnerships with community members such as civic leaders and child-care providers to make changes that promote healthy eating and active living; 2) making it easier for families with children to buy healthy, affordable foods and beverages in their neighborhood; and 3) providing opportunities for children to play safely through access to community parks and other recreation areas.
- Additional information is available at http://www.cdc.gov/vitalsigns.

## Figures and Tables

**TABLE 1:** Distribution of study population, by age, sex, and race/ethnicity — Pediatric Nutrition Surveillance System, United States, 2008 and 2011

Characteristic	2008		2011		p-value*
	
	No.	(%)	No.	(%)	
**Age (yrs)**					
2	1,047,733	(38.1)	1,041,948	(36.2)	
3	888,399	(32.3)	946,112	(32.9)	<0.001
4	811,701	(29.5)	889,408	(30.9)	
**Sex**					
Boys	1,386,995	(50.5)	1,456,503	(50.6)	0.001
Girls	1,360,838	(49.5)	1,420,965	(49.4)	
**Race/Ethnicity**					
Non-Hispanic, white	972,628	(36.9)	989,639	(36.1)	
Non-Hispanic, black	532,968	(20.2)	572,159	(20.9)	
Hispanic	1,030,325	(39.1)	1,069,255	(39.0)	<0.001
American Indian/Alaska Native	24,362	(0.9)	25,224	(0.9)	
Asian/Pacific Islander	77,872	(2.9)	85,475	(3.1)	

* p-value of chi-square test for the difference in the distribution of the demographic characteristics from 2008 to 2011.

**TABLE 2:** Estimated annual change in odds of obesity among low-income children aged 2–4 years, by state/territory — Pediatric Nutrition Surveillance System, United States, 2008–2011*

State/Territory	2008		2009		2010		2011		Crude OR	(95% CI)	AOR†	(95% CI)
			
	No.	%	No.	%	No.	%	No.	%				
Alabama	56,813	13.8	60,572	14.4	65,914	14.1	67,246	14.1	1.01	(1.00–1.02)	0.99	(0.98–1.00)
Arkansas	38,591	13.9	42,270	14.2	32,615	14.1	42,455	14.2	1.01	(1.00–1.02)	1.01	(0.99–1.02)
Arizona	75,338	14.6	85,020	14.3	87,897	14.2	86,516	14.5	1.00	(0.99–1.00)	1.00	(0.99–1.01)
California	301,643	17.3	332,663	17.0	282,216	17.3	260,314	16.8	0.99	(0.99–1.00)	0.99§	(0.99–1.00)§
Colorado	43,476	9.4	51,659	9.0	52,292	9.1	27,467	10.0	1.02	(1.00–1.03)	1.02§	(1.00–1.04)§
Connecticut	25,623	15.5	28,432	16.0	28,401	15.8	27,561	15.8	1.00	(0.99–1.02)	1.00	(0.98–1.01)
District of Columbia	6,195	13.3	6,742	13.6	6,954	13.7	6,940	13.1	1.00	(0.96–1.03)	0.97	(0.94–1.00)
Florida	209,671	14.1	238,542	13.7	242,399	13.4	240,022	13.1	0.97	(0.97–0.98)	0.97§	(0.96–0.97)§
Georgia	124,533	14.8	134,173	14.2	136,379	13.5	138,622	13.2	0.96	(0.95–0.96)	0.95§	(0.95–0.96)§
Hawaii	16,106	9.3	17,252	9.3	17,827	9.1	17,819	9.2	0.99	(0.97–1.02)	0.99	(0.97–1.01)
Iowa	33,548	15.1	36,225	15.0	35,783	14.7	34,327	14.4	0.98	(0.97–0.99)	0.98§	(0.97–0.99)§
Idaho	20,081	12.3	22,620	11.9	22,973	11.4	22,238	11.5	0.97	(0.95–0.99)	0.97§	(0.95–0.99)§
Illinois	121,608	14.7	133,023	14.6	135,408	14.6	132,671	14.7	1.00	(0.99–1.01)	1.00	(0.99–1.01)
Indiana	66,499	14.5	75,671	14.3	78,634	14.2	73,247	14.3	1.00	(0.99–1.00)	0.99	(0.98–1.00)
Kansas	34,352	13.3	36,956	13.2	37,838	13.0	37,419	12.7	0.98	(0.97–1.00)	0.98§	(0.96–0.99)§
Kentucky	62,832	15.7	68,450	15.8	75,189	15.6	33,008	15.5	0.99	(0.98–1.01)	0.99	(0.98–1.00)
Massachusetts	59,297	16.7	63,567	16.8	60,433	16.1	61,094	16.4	0.99	(0.98–1.00)	0.98§	(0.97–0.99)§
Maryland	54,866	15.7	62,194	15.8	63,951	15.7	64,773	15.3	0.99	(0.98–1.00)	0.98§	(0.97–0.99)§
Michigan	103,523	13.9	114,489	13.7	106,019	13.3	115,608	13.2	0.98	(0.97–0.99)	0.97§	(0.97–0.98)§
Minnesota	65,607	13.4	68,997	13.1	68,594	12.7	70,353	12.6	0.98	(0.97–0.99)	0.97§	(0.96–0.98)§
Missouri	60,908	13.9	60,150	13.9	67,547	13.6	67,650	12.9	0.97	(0.96–0.98)	0.97§	(0.96–0.98)§
Mississippi	44,807	14.6	51,741	13.9	52,112	13.7	47,494	13.9	0.98	(0.97–0.99)	0.98§	(0.96–0.99)§
Montana	10,428	12.4	10,105	12.5	8,958	12.2	10,681	11.7	0.98	(0.95–1.00)	0.98	(0.95–1.00)
North Carolina	96,381	15.7	104,323	15.2	105,392	15.5	103,565	15.4	1.00	(0.99–1.00)	0.99	(0.99–1.00)
North Dakota	6,551	13.8	6,968	14.1	6,836	14.1	6,665	13.1	0.98	(0.95–1.01)	0.99	(0.95–1.02)
Nebraska	20,658	13.9	20,811	14.2	22,194	13.8	22,136	14.3	1.01	(0.99–1.02)	1.00	(0.99–1.02)
New Hampshire	8,082	15.5	8,963	14.4	8,621	14.2	8,219	14.6	0.98	(0.95–1.00)	0.97§	(0.94–0.99)§
New Jersey	68,163	17.9	75,191	18.4	78,181	17.3	77,476	16.6	0.97	(0.96–0.97)	0.96§	(0.95–0.97)§
New Mexico	22,295	12.0	31,433	12.0	31,043	11.7	30,269	11.3	0.98	(0.96–0.99)	0.97§	(0.96–0.99)§
Nevada	23,348	12.9	28,159	13.9	32,080	13.6	33,427	12.7	0.99	(0.97–1.00)	0.99	(0.98–1.01)
New York	209,713	14.6	224,130	14.4	224,243	14.5	229,291	14.3	1.00	(0.99–1.00)	0.99§	(0.99–1.00)§
Ohio	125,011	12.2	130,792	12.3	128,754	12.4	121,624	12.4	1.01	(1.00–1.01)	1.00	(0.99–1.01)
Oregon	49,193	14.7	52,713	15.0	54,150	15.1	54,212	14.9	1.00	(0.99–1.02)	1.01	(1.00–1.02)
Pennsylvania	111,767	11.5	119,134	12.0	117,337	12.0	119,812	12.2	1.02	(1.01–1.03)	1.02§	(1.01–1.03)§
Puerto Rico	99,828	17.9	100,313	18.1	89,438	18.3	89,463	17.9	1.00	(0.99–1.01)	1.00	(0.99–1.01)
Rhode Island	11,466	16.2	12,456	16.2	12,922	15.5	12,644	16.6	1.00	(0.98–1.02)	1.00	(0.98–1.02)
South Dakota	9,125	16.2	9,705	16.4	10,106	16.1	10,312	15.2	0.97	(0.95–1.00)	0.97§	(0.95–1.00)§
Tennessee	69,015	13.5	71,914	14.0	71,349	14.5	69,276	14.2	1.02	(1.01–1.03)	1.02§	(1.01–1.03)§
U.S. Virgin Islands	2,339	13.6	2,587	11.9	2,587	11.2	2,552	11.0	0.92	(0.88–0.98)	0.92§	(0.87–0.97)§
Vermont	7,009	13.3	7,051	13.2	6,921	12.2	6,168	12.9	0.98	(0.95–1.01)	0.98	(0.95–1.01)
Washington	92,980	14.4	104,389	14.4	105,886	14.4	106,346	14.0	0.99	(0.98–1.00)	0.99§	(0.98–1.00)§
Wisconsin	55,875	13.6	60,280	13.7	59,975	14.1	58,745	14.0	1.01	(1.00–1.02)	1.01	(1.00–1.02)
West Virginia	22,689	13.5	23,739	13.4	23,205	13.7	22,581	14.0	1.02	(1.00–1.03)	1.02	(1.00–1.03)

**Abbreviations:** OR = odds ratio; AOR = adjusted odds ratio; CI = confidence interval.

* Includes 43 states and territories with consistent data during 2008–2011.

† Estimated annual change in odds of obesity, calculated from logistic regression models controlling for age, sex, and race/ethnicity.

§ Statistically significant at p<0.05; some 95% CIs include 1.00 because of rounding.

## Footnotes

* Dalenius K BE, Smith B, Polhamus B, Grummer-Strawn L. Pediatric nutrition surveillance 2010 report. Atlanta, GA: US Department of Health and Human Services, CDC; 2012.

† CDC. Pediatric Nutrition Surveillance Sytem. Atlanta, GA: US Department of Health and Human Services, 2013. Available at http://www.cdc.gov/pednss/pednss_tables/pdf/national_table12.pdf.

§ Kuczmarski RJ, Ogden CL, Guo SS, et al. 2000 CDC growth charts for the United States: methods and development. Vital Health Stat 11 2002;(246). Available at http://www.cdc.gov/nchs/data/series/sr_11/sr11_246.pdf.

¶ Additional information available at http://www.cdc.gov/nccdphp/dnpa/growthcharts/resources/biv-cutoffs.pdf.

** Additional information available at http://www.letsmove.gov/mayors-local-officials.

†† Additional information available at http://www.letsmove.gov/white-house-task-force-childhood-obesity-report-president.
